# Catheter-Associated Urinary Tract Infections, Bacteremia, and Infection Control Interventions in a Hospital: A Six-Year Time-Series Study

**DOI:** 10.3390/jcm11185418

**Published:** 2022-09-15

**Authors:** Amalia Papanikolopoulou, Helena C. Maltezou, Athina Stoupis, Dimitra Kalimeri, Androula Pavli, Fotini Boufidou, Maria Karalexi, Nikos Pantazis, Constantinos Pantos, Yannis Tountas, Vasiliki Koumaki, Maria Kantzanou, Athanasios Tsakris

**Affiliations:** 1 Clinical Pharmacology Department, Athens Medical Center, 5-7 Distomou Str., Marousi, 15125 Athens, Greece; 2 Directorate of Research, Studies, and Documentation, National Public Health Organization, 3-5 AgrafonStr., Marousi, 15123 Athens, Greece; 3 Clinical Infectious Diseases Department, Athens Medical Center, 58 Kifissias Avenue, Marousi, 15125 Athens, Greece; 4 Nurse Department Athens Medical Center, 5-7 Distomou Str., Marousi, 15125 Athens, Greece; 5 Department of Travel Medicine, National Public Health Organization, 3-5 Agrafon Str., Marousi, 15123 Athens, Greece; 6 Neurochemistry and Biological Markers Unit, 1st Department of Neurology, School of Medicine, Eginition Hospital, National and Kapodistrian University of Athens, 11528 Athens, Greece; 7 Department of Microbiology, Faculty of Medicine, School of Health Sciences, National and Kapodistrian University of Athens, 75 Mikras Asias Str., 15772 Athens, Greece; 8 Department of Hygiene, Epidemiology and Medical Statistics, Faculty of Medicine, School of Health Sciences, National and Kapodistrian University of Athens, 75 Mikras Asias Str., 15772 Athens, Greece; 9 Department of Pharmacology, Faculty of Medicine, School of Health Sciences, National and Kapodistrian University of Athens, 75 Mikras Asias Str., 15772 Athens, Greece

**Keywords:** catheter-associated urinary tract infection, healthcare-associated infections, infection control measures, time series data, multi-drug resistant bacteria

## Abstract

Catheter-associated urinary tract infections (CAUTIs) are among the most common healthcare-associated infections. Urine catheters are often reservoirs of multidrug-resistant (MDR) bacteria and sources of pathogens transmission to other patients. The current study was conducted to investigate the correlation between CAUTIs, MDR bacteremia, and infection control interventions, in a tertiary-care hospital in Athens, from 2013 to 2018. The following data were analyzed per month: 1. CAUTI incidence; 2. consumption of hand hygiene disinfectants; 3. incidence of isolation of MDR carrier patients, and 4.incidence of bacteremia/1000 patient-days [total resistant a.Gram-negative: carbapenem-resistant *Pseudomonas aeruginosa*, *Acinetobacter baumannii*, and *Klebsiella pneumoniae*; b.Gram-positive: vancomycin-resistant Enterococci and methicillin-resistant Staphylococcus aureus]. The use of scrub disinfectant solutions was associated with decreased CAUTI rate in Total Hospital Clinics (OR: 0.97, 95% CI: 0.96–0.98, *p*-value: <0.001) and in Adults ICU (OR: 0.79, 95% CI: 0.65–0.96, *p*-value:0.018) while no correlation was found with isolation rate of MDR-carrier pathogens. Interestingly, an increase in total bacteremia (OR: 0.81, 95% CI: 0.75–0.87, *p*-value:<0.001) or carbapenem-resistant bacteremia correlated with decreased incidence of CAUTIs (OR: 0.96, 95% CI: 0.94–0.99, *p*-value: 0.008). Hand hygiene measures had a robust and constant effect on infection control, reducing the incidence of CAUTIs.

## 1. Introduction

Urinary tract infection (UTI) is one of the most common healthcare-associated infections (HAIs), and approximately two-thirds of these infections are attributed to an indwelling urethral catheter [1]. The first pan-European point prevalence survey of HAIs in 2011–2012 found that UTIs accounted for 19% of all HAIs in acute-care hospitals in Europe [2]. Catheter-associated urinary tract infection (CAUTI) has been associated with increased morbidity, mortality, hospital cost, and length of stay [3,4,5]. In addition, bacteriuria commonly leads to unnecessary antimicrobial use, and urinary drainage systems are often reservoirs of multidrug-resistant (MDR) bacteria and sources of transmission of pathogens to other patients [6,7]. The source of microorganisms causing CAUTI can be endogenous, typically via meatal, rectal, or vaginal colonization, or exogenous, such as via contaminated hands of healthcare personnel or equipment [8].

The most prevalent pathogen associated with CAUTI in hospital wards and ICUs are *Escherichia coli*, followed by *Klebsiella* spp., *Enterococcus* spp., *Pseudomonas aeruginosa*, and *Enterobacter* spp. [9]. In addition, approximately one third of *E. coli* isolates and a quarter of *P. aeruginosa* isolates from CAUTI cases are fluoroquinolone-resistant, while resistance to other advanced class antibiotics such as third-generation cephalosporins and carbapenems, and MDR pathogens are also substantial [10].

The correlation between HAIs and infection control measures has been studied n the last decade [11,12,13]. More recent studies using interrupted time series analysis, focused on evaluating educational and interventional bundles to reduce CAUTIs [14,15,16]. The aim of the current prospective study was to investigate the correlation between the incidence rate of CAUTIs, specific infection control measures, and the incidence rate of MDR bacteremia in a hospital in Greece.

## 2. Materials and Methods

### 2.1. Setting

The study was conducted prospectively from January 2013 to December 2018 in a 300-bed private tertiary-care hospital in Athens. The hospital consists of: 1. one Adults Clinic with Internal Medicine, Oncology, Hematology, Surgery Departments and one ICU; 2. one Obstetrics and Gynecology Clinic with one neonatal ICU, and 3. one Pediatrics Clinic with one pediatric ICU.

### 2.2. Infection Control Measures

As we already described [17,18], the following infection control measures were applied: 1. active surveillance of carbapenem-resistant *A. baumannii*, *P. aeruginosa*, and *K. pneumoniae*, and methicillin-resistant *Staphylococcus aureus* (*MRSA*) and vancomycin-resistant *Enterococci* (*VRE*); 2. implementation of a CAUTI bundle, which consisted of aseptic insertion and maintenance techniques, catheter change guidelines, and discontinuation indications; 3. promotion of hand hygiene before and after providing healthcare to patients; 4. carriage screening (pharyngeal, axillary-rectal, nasal) cultures followed by isolation of MDR carrier patients; and 5. audit of CAUTI bundle and ASP on a monthly basis.

### 2.3. Data Collection

Data were collected prospectively on a monthly basis: 1. number of CAUTIs was collected from Clinical Infectious Diseases Department; 2. catheterization was detected and recorded manually by clinical visits from Nurse Department; 3. hand disinfectant solutions use was collected from Pharmacy Department; 4. number of bacteremia was collected from Microbiology Department. The medical/nurse hospital quality procedures and laboratory diagnostic procedures were supervised by Quality Assurance Department and did not change throughout the six year-study period.

### 2.4. Outcomes

Estimated outcomes included: 1. CAUTI rate (incidence/1000 catheter-days); 2. consumption of hand disinfectant solutions (L/1000 patient-days); and 3. incidence of bacteremia (incidence/1000 patient-days). The outcomes were estimated on a monthly basis.

### 2.5. Detection of Bacteremia

Bacteremia was detected through Gram stains and blood cultures. The automated VITEK 2 system (Biomerieux) was used for isolation, identification, and antibiotic susceptibility testing. The CLSI breakpoints were applied. The susceptibility of bacteria was determined by Kirby–Bauer test, MIC semi-automated testing, and/or E-test.

### 2.6. Definitions

CAUTI in a patient with an indwelling urethral catheter is defined as the onset of signs and/or symptoms compatible with UTI and no other source of infection along with ≥10^3^ colony-forming units (cfu)/mL of ≥1 bacterial species in a single urine specimen or in a midstream voided urine specimen from a patient whose catheter was removed the past 48 h [19]. Signs and symptoms compatible with CAUTI include new onset or worsening of fever, rigors, altered mental status or lethargy with no other identified cause; costovertebral angle tenderness; acute hematuria; pelvic discomfort; and in those whose catheters have been removed, dysuria, urgent or frequent urination, or suprapubic pain or tenderness [19]. Isolation rate of MDR-carrier patients was expressed as a percentage of isolated patients per 100 admissions. Bacteremia was defined as a laboratory-confirmed bloodstream infection, either primary (not related to an infection at another body site) or secondary (thought to be seeded from a site-specific infection at another body site) [20,21]. New episode of bacteremia within a month period was defined as a new episode of bacteremia due to a different pathogen strain or due to the same pathogen strain but with different phenotype of resistance. The incidence of total bacteremia is the sum of total Gram-positive and Gram-negative bacteremia. The incidence of total carbapenem-resistant Gram-negative bacteremia refers to carbapenem-resistant *A. baumannii*, *P. aeruginosa* and *K. pneumoniae* bacteremia. The incidence of total resistant Gram-positive bacteremia refers to the incidence of MRSA and VRE bacteremia [21]. Hand hygiene concerns scrub disinfectant solutions with chlorhexidine, alcohol 70% disinfectant solutions with chlorhexidine, and/or simple soap [18].

### 2.7. Statistical Analysis

As already described [17,18], an analysis of time trends in the intervention and outcome variables during the study period was initially performed. The variable under investigation was the dependent variable. Time since the beginning of the study (in months) was the independent variable in the regression models and was entered through appropriate restricted cubic splines. In order to capture potential seasonality effects, Fourier series terms of time (1st and 2nd order) were also used in the models. Standard errors (SE) and corresponding 95% confidence intervals (CI) were estimated using the robust (sandwich) variance estimator to adjust for potential violations of models’ assumptions. Estimated values for start and end of the study period and corresponding 95% CIs were estimated through a simplification of the models. Spline time terms were replaced by a single linear time trend or two piecewise linear terms to capture average long-term trend. A linear regression model was applied for consumption of hand disinfectants. When the outcome of interest was CAUTI or bacteremia rates, Poisson regression models were applied with number of cases as dependent variable and the appropriate number of catheter-days or patient-days, respectively, used as an offset after logarithmic transformation [17]. When the percentage over total number of hospitalizations was the outcome of interest (e.g., isolations), binomial regression models were applied with the number of cases as the dependent variable and the appropriate number of hospitalizations as the binomial denominator [17]. Associations between outcomes and interventions were studied by introducing appropriate independent variables into the models [22]. The effects of the independent variables were initially tested separately for current (“month 0”) and lagged values (months −1, −2 and −3). In case of statistical significance (*p*-value < 0.05) or indicative significance (0.05 < *p*-value < 0.10) for more than one case (e.g., in month 0 and in month −1) and association direction was the same (e.g., positive for both), average value was used as independent variable. In cases where the direction of the association was different (e.g., positive for “month 0” and negative for “month −1”), the results of the respective models are presented separately [22]. All *p*-values reported throughout the manuscript have not been adjusted for multiple testing. Analyses were conducted using Stata version 14.2 (Stata Corporation, College Station, TX, USA).

## 3. Results

**Figure 1:**
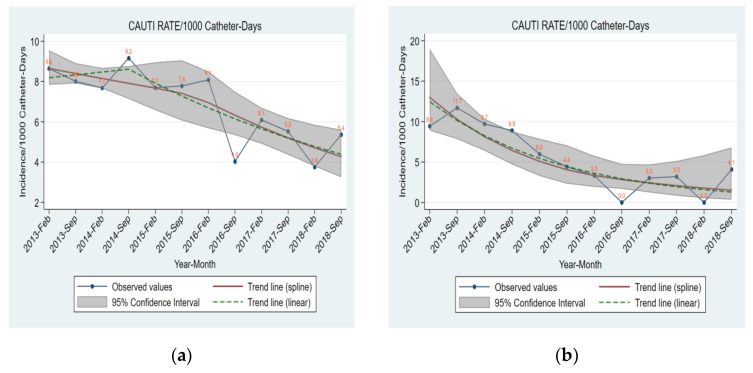
Observed values and estimated time trends for CAUTI rate in (**a**) total Hospital Clinics, (**b**) Adults Intensive Care Unit from January 2013 to December 2018.CAUTI: catheter-associated urinary tract infection; trends shown with dashed lines derived from Poisson regression models with robust standard errors, seasonality terms and linear or piecewise linear long term trend: log(N) = β_0_ +β_1_t_−_ +β_2_t _+_ +β_3_ × sin(2πt/12) + β_4_ × cos(2πt/12) + β_5_ × sin(4πt/12) + β_6_ × cos(4πt/12) + log(ventilator-days) with N being the number of cases and t being time since study start in months (t_−_ and t_+_ piecewise linear time terms). Trends shown with grey area derived similarly but spline terms of time were used for long term trend instead of piecewise linear terms.

A total of 95,228 admissions occurred during the entire study period. Overall, 12.84% of hospitalized patients underwent catheterization. There were 379 CAUTI episodes among 12,228 catheterized patients; therefore, the CAUTI rate during the six-year study period was 5.28 episodes of CAUTI/1000 catheter days. The monthly CAUTI incidence in all Hospital Clinics and in Adults ICU are depicted in Figure 1. Results over time for each measure are shown in Table 1, Table 2, Table 3 and Table 4. The relationship between CAUTI rates and concurrent or lagged (1–3 months) values of each process measure are shown in Table 5 and Table 6.

The time trends of CAUTI rate during the six-year study period in the entire hospital and divisions are shown in Table 1. The incidence of CAUTI decreased significantly in all Hospital Clinics and Departments and also in the Adults ICU (*p*-value: <0.001 for all comparisons).

Table 2 shows time trends of incidence of isolations per 100 admissions. A significant increase in the rate of isolation of patients with MDR pathogens was observed in Total Hospital Departments and in Adults ICU (*p*-value = <0.001), while in Adults Clinic the increase was significant up to April 2015 only (*p*-value < 0.001) and in Adults Departments up to February 2015 only (*p*-value < 0.001).

Table 3 shows time trends in the consumption of hand disinfectant solutions per category of disinfectant. There was a statistically significant increase in the consumption of alcohol disinfectant solutions and all hand disinfectant solutions in all Departments and Clinics (*p*-value = <0.001). In Adults Clinic Departments separately the increase was observed only in alcohol disinfectant solutions (*p*-value = <0.001). In the ICU it is noticeable that there was a statistically significant increase in scrub and all hand disinfectant solutions (*p*-value = 0.001). Combining the results with Figure 1b for the ICU, the yearly increase of 24.4% from 2013 up to 8/2016 (95% CI: 16.8–30.1, *p*-value: <0.001) in the consumption of scrub disinfectant solutions agrees with the 1st trough value of CAUTI rate per month in September 2016, and the 6-year increase in the consumption of all hand disinfectant solutions with the 2nd trough value of CAUTI rate per month in February 2018.

Table 4 shows the time trends in the incidence of different bacteremia/1000 patient-days. The incidence of total bacteremia increased significantly in the entire hospital and divisions (*p*-value = <0.001), which is attributed to the increase in the number of blood cultures and admissions. However, there was no statistically significant difference in the incidence of total bacteremia from resistant pathogens. In total Hospital Clinics and Departments, the trend in the incidence of total carbapenem-resistant Gram-negative pathogens decreased not significantly, while in Adults ICU increased not significantly. The analysis per pathogen showed a significant decrease only for carbapenem-resistant *P. aeruginosa* in total Hospital Clinics (*p*-value = 0.027) and Departments (*p*-value = 0.042), and in Adults Clinic (*p*-value = 0.031) and Departments (*p*-value = 0.051). For carbapenem-resistant *K. pneumoniae* and *A. baumannii* the incidence did not change significantly. Interestingly the latter had zero incidence in the hospital departments. The incidence of resistant Gram-positive pathogens remained very low and stable throughout the entireperiod; for this reason, we did not apply a linear model. Finally, in the Adults ICU the analysis per pathogen did not show any statistical change for any of the carbapenem resistant Gram-negative bacteria.

The correlation of CAUTI with the incidence of different bacteremia is shown in Table 5. Every increase in the incidence of total bacteremia the current and the previous month correlated with a decreased CAUTI rate, in total Hospital Clinics and Departments and in Adults Clinics and Departments (*p*-value = <0.001). There was a negative correlation for total carbapenem-resistant Gram-negative bacteremia in total Hospital Clinics (*p*-value = 0.008) and Adults Clinics (*p*-value = 0.042) three months before, but in all Hospital Departments (*p*-value = 0.048) and Adults Departments (*p*-value = 0.060), we noticed a positive correlation for current month and negative three months before (*p*-value = 0.009 and 0.027, respectively). For each category of carbapenem-resistant Gram-negative pathogen, the negative correlation was constant in all Hospital Clinics/Departments and in Adults Clinics/Departments, and statistically significant especially for carbapenem-resistant *K. pneumoniae* bacteremia (*p*-value < 0.001). Every increase in their incidence two and three months earlier resulted in a decreased CAUTI rate. Every increase in the incidence of carbapenem-resistant *P. aeruginosa* bacteremia two and three months earlier significantly correlated with increased CAUTI rate (*p*-value < 0.001). This phenomenon was not repeated for the other Gram-negative resistant pathogens. However, there was a significant correlation between the incidence of carbapenem-resistant *K. pneumoniae* and a decrease in CAUTI incidence three months earlier (*p*-value = 0.018).

Table 6 shows the correlation between CAUTI and infection control measures. Every increase in the consumption of either scrub or all disinfectant solutions the previous months significantly correlated with decreased CAUTI rate in all Hospital Clinics (*p*-value = <0.001 and 0.004), Adults Clinic (*p*-value = <0.001 and 0.006), and Adults Departments (*p*-value = <0.001 and 0.005). In the Adults ICU, every increase in the consumption of alcohol and all disinfectant solutions current month correlated significantly with increased CAUTI (*p*-value = 0.012 and 0.016), while every increase the previous month in the consumption of scrub disinfectant solutions correlated significantly with decreased CAUTI rate (*p*-value = 0.018). Finally, the intervention of isolation of patients did not show a direct correlation with the CAUTI rate.

## 4. Discussion

In this six-year study we studied the relationship among infection control measures and outcomes with CAUTI in a tertiary-care hospital located in Athens. In our institution from 2013 to 2018 a CAUTI-bundle was implemented to promote the rational use of indwelling urinary catheters, resulting overall in a catheterization percentage of 12.84%, which stands within international references [23,24]. According to the 2011–2012 pan-European point prevalence survey of HAIs, the mean catheterization rate of hospitalized patients in acute care hospitals in European countries was 17.2%, while in Greece it was 30% [2]. In our study, the 6-year CAUTI rate was 5.27 infections per 1000 catheter days, which is within the range of pooled mean CAUTI rates (3.1–7.5 infections per 1000 catheter-days) found in acute care hospitals during 2015–2017, as reported by National Healthcare Safety Network [9].

During the last 15 years many guidelines for the prevention of CAUTI have been published [19,25,26,27], and many programs with evidence-based educational and interventional bundles to reduce the incidence of CAUTI have been evaluated [28,29,30,31,32,33,34,35,36]. In our study a CAUTI-bundle was implemented along with other infection control interventions, resulting in a significant reduction in the incidence of CAUTI in all hospital Clinics and Departments, and in Adults ICU.

In our hospital, the most significant infection control interventions were the increased isolation of MDR carrier patients and the increased consumption of hand disinfectant solutions indicating adherence to hand hygiene. Furthermore, the most significant outcome was the decrease in the CAUTI rate. The decrease in the incidence of carbapenem-resistant Gram-negative bacteremia, either in total or per studied pathogen, although it was indicative, reflects the effect of isolation of patients with MDR pathogens, while very low or zero incidence of resistant Gram-positive pathogens during the six-year study period, reflects the effect of implementation of hand hygiene [17,21]. Our findings, in different clinics and departments of the hospital, depict the continuous need of infection control interventions with tailored frequency, evaluation, and implementation.

Until recently, it was more feasible to design a study regarding bundled horizontal infection prevention strategies for the prevention of HAI [28,29,30,31,32,33,34]. In our study, for the first time in the literature, we have results regarding the association of CAUTI with different bacteremia and with consumption of hand disinfectant solutions for the entire hospital as well as its Clinics and Departments separately. For CAUTI and total bacteremia, the correlation was always negative and significant, indicating no cross-infection between blood and UTIs. In case of total carbapenem-resistant Gram-negative bacteremia, the correlation was positive only for the current month, in Total Hospital Departments, showing the severity of such infections. For each category of carbapenem-resistant Gram-negative pathogens, the correlation with CAUTI was negative, in the entire hospital and its divisions, two and three months earlier, showing a preserve time effect of the implemented interventions. For Adults ICU the results diverged from one carbapenem-resistant bacterium to another. Especially for carbapenem-resistant *P. aeruginosa* bacteremia the correlation with increased CAUTI rate, two and three months earlier, showed a disability of the implemented interventions to have prolonged effect for this type of pathogen, and the need for further tailored infection control interventions regarding this hydrophilic Gram-negative bacterium. Interestingly, the incidence of carbapenem-resistant *A. baumannii* and *K. pneumoniae* correlated with the decreased CAUTI rate, indicating different infection control behavior of these pathogens.

From the results of CAUTI and hand disinfectant solutions, the correlation was negative in the entire hospital and its divisions giving a 2 to 3 month-lasting effect of implementation of hand hygiene. While no actual changes in catheter indwelling rates and indwelling duration was noted (data not shown), CAUTI-bundle education regarding hand sanitizer consumption resulted in decreased CAUTI rate in the hospital setting. Especially in the ICU both trough values of CAUTI rate coincide with the increase consumption of scrub and all hand disinfectant solutions implying the importance of hand washing along with disinfection in controlling nosocomial infections such as CAUTI.

Moreover, for the first time in the literature, the correlation of CAUTI with the isolation of MDR-carrier patients was investigated and non-significance was found. Worthy to mention though that both decrease in CAUTI and increase in isolation of patient directly correlated with decrease in incidence of MDR bacteremia.

Our study has several strengths. For the first a time series analysis was used to study the potential association among CAUTI rate, infection control measures, and MDR bacteremia. A clear strength is the prospective study design and the prolonged study period. The analysis of findings per clinic and department gave us the opportunity to end up with more accurate conclusions. Catheter-associated asymptomatic bacteriuria was not included in the definition of CAUTI, which is a potential limitation. Lastly, since the analysis of data included the study of associations between various outcomes and potential predictors in several clinics, several hypotheses were investigated. Some inflation of the Type I error beyond the typical 0.05 level may be considered, since we selected to present unadjusted *p*-values [37].

## 5. Conclusions

We prospectively studied the association of infection control measures and CAUTI incidence. The consumption of all hand disinfectant solutions and scrub correlated with decreased CAUTI rate in total Hospital and its divisions, while no correlation was found with the intervention of isolation of MDR carrier patients. Moreover, the correlation of CAUTI with MDR bacteremia was investigated and was found always negative, which indicates a constant and robust effect of the infection control program. Time series analysis can be used to inform evidence-based interventions and infection control policies.

## Figures and Tables

**Table 1:** Time trend of CAUTI rate in a hospital from January 2013 to December 2018.

Time Trend					
CAUTI Rate Per Month	EVSP Jan 2013 (95% CI)	EVEP Dec 2018 (95% CI)	*p*-Value	% Relative Change/Year (95% CI)	*p*-Value
**Total Hospital Clinics**	8.2 (7.4 to 9.0)	4.4 (3.5 to 5.5)	<0.001	3.5 (−9.0 to 17.6) up to September 2014	0.604
				−15.5 (−21.1 to −9.5) after September 2014	<0.001
**Total Hospital Departments**	6.8 (5.1 to 9.0)	5.4 (5.0 to 5.9)	<0.001	13.9 (−0.9 to 30.9) up to September 2015	0.067
				−16.9 (−21.5 to −12.0) after September 2015	<0.001
**Adults Clinic**	8.5 (7.7 to 9.4)	4.4 (3.5 to 5.5)	<0.001	3.0 (−9.5 to 17.0) up to September 2014	0.657
				−18.2 (−24.3 to −12.5) after September 2014	<0.001
**Adults Clinic Departments**	7.1 (5.3 to 9.4)	5.4 (5.0 to 5.9)	<0.001	13.2 (−1.9 to 30.6) up to September 2015	0.090
				−17.5 (−22.3 to −12.4) after September 2015	<0.001
**Adults ICU**	12.5 (9.1 to 17.0)	1.3 (0.5 to 3.5)	<0.001	−33.8(−46.8 to −17.7)	<0.001

CAUTI: catheter-associated urinary tract infection; ICU: intensive care unit; EVSP: estimated value start period; EVEP: estimated value end period; CI: confidence interval. All estimates derived from Poisson regression models with robust standard errors, seasonality terms and linear or piecewise linear long-term trend: log(N) = β_0_ +β_1_t_−_ +β_2_t _+_ +β_3_ × sin(2πt/12) + β_4_ × cos(2πt/12) + β_5_ × sin(4πt/12) + β_6_ × cos(4πt/12) + log(catheter-days) with N being the number of cases and t being time since study start in months (t_−_ and t_+_ piecewise linear time terms; when piecewise linear long term trend was not required a single time term was used). % Relative changes/year derived as [exp(12 × β_1_,_2_)−1] × 100%.

**Table 2:** Time trend of isolations/100 hospital admissions in a hospital from January 2013 to December 2018.

Time Trend					
% Isolations/Admissions	EVSP Jan 2013 (95% CI)	EVEP Dec 2018 (95% CI)	*p*-Value	% Relative Change/Year (95% CI)	*p*-Value
**Total Hospital Clinics**	8.0 (7.3 to 8.8)	5.6 (5.2 to 6.0)	<0.001	−2.2 (−6.2 to 1.8) up to April 2016	0.276
				−11.0 (−14.7 to −7.1) after April 2016	<0.001
**Total Hospital Departments**	1.0 (0.6 to 1.6)	2.5 (2.1 to 3.0)	<0.001	86.6 (46.5 to 137.7) up to March 2015	<0.001
				−9.6 (−15.7 to −3.1) after March 2015	0.005
**Adults Clinic**	4.1 (3.4 to 4.9)	5.0 (4.5 to 5.6)	0.059	24.0 (12.3 to 36.9) up to April 2015	<0.001
				−6.9 (−11.3 to −2.4) after April 2015	0.003
**Adults Clinic Departments**	1.7 (1.1 to 2.5)	2.6 (2.2 to 3.0)	0.053	59.8 (28.7 to 98.5) up to February 2015	<0.001
				−13.1 (−18.5 to −7.4) after February 2015	<0.001
**Adults ICU**	20.4 (19.2 to 21.8)	27.6 (24.1 to 31.4)	<0.001	14.4 (10.6 to 18.2) up to June 2017	<0.001
				−12.3 (−24.8 to 2.1) after June 2017	0.091

ICU: intensive care unit; EVSP: estimated value start period; EVEP: estimated value end period; CI: confidence interval1; All estimates derived from binomial logistic regression models with robust standard errors, seasonality terms and piecewise linear long term trend: logit(π) = β_0_ +β_1_t_−_ +β_2_t _+_ +β_3_ × sin(2πt/12) + β_4_ × cos(2πt/12) + β_5_ × sin(4πt/12) + β_6_ × cos(4πt/12) with π being theprobability of isolation and t being time since study start in months (t_−_ and t_+_ piecewise linear time terms). % Relative changes/year derived as [exp(12 × β_1_,_2_)−1] × 100%.

**Table 3:** Time trend of hand disinfectant solutions use in a hospital from January 2013 to December 2018.

Time Trend					
Hand Disinfectant Sol Consumption L/1000 Patient-Days	EVSP Jan 2013 (95% CI)	EVEP Dec 2018 (95% CI)	*p*-Value	% Relative Change/Year (95% CI)	*p*-Value
Total Hospital Clinics					
Alcohol disinfectant sol	26.0	48.7	<0.001	16.2 (12.4 to 20.0) up to September 2014	<0.001
	(21.7 to 30.2)	(46.8 to 50.6)		−1.0 (−2.2 to 0.2) after September 2014	0.100
Scrub disinfectant sol	29.7	27.9	0.321	3.4 (0.6 to 6.2) up to October 2014	0.020
	(25.9 to 33.4)	(26.4 to 29.3)		−1.9 (−2.6 to −1.1) after October 2014	<0.001
Simple soap sol	15.8	9.7	<0.001	−3.9 (−5.0 to −2.7) up to October 2014	<0.001
	(14.5 to 17.2)	(8.3 to 11.1)		0.2 (−0.4 to 0.7) after October 2014	0.542
All hand disinfectant sol	71.5	86.4	0.001	15.7 (8.8 to 22.6) up to September 2014	<0.001
	(62.6 to 80.3)	(83.6 to 89.2)		−2.7 (−4.3 to −1.0) after September 2014	0.002
**Total Hospital Departments**					
Alcohol disinfectant sol	16.0	64.6	<0.001	26.4 (22.8 to 30.0) up to October 2014	<0.001
	(12.2 to 19.8)	(60.8 to 68.4)		0.6 (−1.1 to 2.3) after October 2014	0.486
Scrub disinfectant sol	27.7	27.1	0.733	1.1 (0.4 to 1.8) up to December 2017	0.002
	(25.5 to 29.8)	(24.5 to 29.7)		−6.0 (−9.8 to −2.3) after December 2017	0.002
Simple soap sol	19.0	12.9	<0.001	−3.7 (−5.0 to −2.5) up to October 2014	<0.001
	(17.7 to 20.2)	(11.1 to 14.6)		0.1 (−0.5 to 0.7) after October 2014	0.749
All hand disinfectant sol	62.3	109.5	<0.001	25.0 (19.4 to 30.6) up to September 2014	<0.001
	(57.2 to 67.5)	(103.3 to 115.6)		1.3 (−1.2 to 3.8) after September 2014	0.309
**Adults Clinic**					
Alcohol disinfectant sol	34.6	34.3	0.870	15.4 (11.1 to 19.7) up to July 2014	<0.001
	(32.1 to 37.2)	(31.2 to 37.5)		−5.3 (−6.9 to −3.7) after July 2014	<0.001
Scrub disinfectant sol	37.3	33.2	0.057	−0.7 (−1.4 to 0.0)	0.057
	(34.3 to 40.3)	(31.3 to 35.0)			
Simple soap sol	22.6	13.9	<0.001	−5.4 (−6.9 to −3.9) up to November 2014	<0.001
	(21.2 to 24.0)	(11.5 to 16.2)		0.3 (−0.6 to 1.2) after November 2014	0.528
All hand disinfectant sol	91.0	79.7	<0.001	12.3 (5.7 to 18.8) up to July 2014	<0.001
	(85.3 to 96.7)	(75.5 to 83.8)		−6.7 (−8.8 to −4.7) after July 2014	<0.001
**Adults Clinic Departments**					
Alcohol disinfectant sol	25.7	46.3	<0.001	20.2 (16.5 to 24.0) up to August 2014	<0.001
	(22.3 to 29.2)	(43.9 to 48.6)		−2.7 (−3.9 to −1.4) after August 2014	<0.001
Scrub disinfectant sol	40.1	32.9	0.004	−2.5 (−3.7 to −1.2) up to September 2016	<0.001
	(36.5 to 43.7)	(29.7 to 36.1)		0.8 (−1.4 to 3.1) after September 2016	0.463
Simple soap sol	25.5	13.1	<0.001	−6.3 (−8.1 to −4.5) up to October 2014	<0.001
	(23.9 to 27.1)	(10.5 to 15.6)		−0.3 (−1.3 to 0.6) after October 2014	0.468
All hand disinfectant sol	88.1	88.6	0.901	13.7 (6.8 to 20.5) up to August 2014	<0.001
	(80.4 to 95.8)	(84.5 to 92.7)		−4.9 (−6.8 to −3.0) after August 2014	<0.001
**Adults ICU**					
Alcohol disinfectant sol	98.0	83.2	0.286	−2.5 (−7.2 to 2.1)	0.286
	(80.9 to 115.1)	(68.5 to 97.9)			
Scrub disinfectant sol	1.9	35.9	0.001	24.4 (18.6 to 30.1) up to August 2016	<0.001
	(7.4 to 11.3)	(17.7 to 54.2)		−22.9 (−34.4 to −11.4) after August 2016	<0.001
All hand disinfectant sol	117.0	179.3	0.001	10.5 (4.6 to 16.5)	0.001
	(97.3 to 136.7)	(157.8 to 200.9)			

ICU: intensive care unit; L: liter; sol: solution; EVSP: estimated value start period; EVEP: estimated value end period; CI: confidence interval; All estimates derived from linear regression models with robust standard errors, seasonality terms and piecewise linear long term trend: E[Y] = β_0_ + β_1_t_−_ +β_2_t _+_ +β_3_ × sin(2πt/12) + β_4_ × cos(2πt/12) + β_5_ × sin(4πt/12) + β_6_ × cos(4πt/12) with E[Y] being theexpected consumption value and t being time since study start in months (t_−_ and t_+_ piecewise linear time terms). Absolute changes/year derived as β_1_,_2_ × 12.

**Table 4:** Time trend of the incidence of bacteremia in a hospital from January 2013 to December 2018.

Time Trend					
Incidence of Bacteremia/1000 Patient-Days	EVSP Jan 2013 (95% CI)	EVEP Dec 2018 (95% CI)	*p*-Value	% Relative Change/(95% CI)	*p*-Value
Total Hospital Clinics					
Total Bacteremia	3.4 (3.0 to 3.8)	5.0 (4.5 to 5.5)	<0.001	2.4 (−2.2 to 7.1) up to December 2016	0.311
				15.9 (6.7 to 25.9) after December 2016	<0.001
Total Bacteremia MDR Gram(+) and (−)	0.4 (0.3 to 0.5)	0.2 (0.2 to 0.3)	0.093	−8.0 (−16.5 to 1.4)	0.093
Total Bacteremia CR Gram(−)	0.3 (0.2 to 0.5)	0.2 (0.1 to 0.3)	0.099	−7.8 (−16.2 to 1.5)	0.099
Total Bacteremia MDR Gram(+)	0.0 (0.0 to 0.1)	0.0 (0.0 to 0.1)	0.467	−8.5 (−28.0 to 16.3)	0.467
Total Bacteremia CR-Ac	0.1	0.1	0.935	−46.7 (−67.6 to −12.3) up to March 2016	0.013
	(0.0 to 0.2)	(0.0 to 0.2)		102.3 (10.9 to 269.0) after March 2016	0.022
Total Bacteremia CR-KlPn	0.1	0.0	0.545	51.2 (−10.0 to 153.8) up to February 2015	0.118
	(0.0 to 0.2)	(0.0 to 0.1)		−27.1 (−43.0 to −6.8) after February 2015	0.012
Total Bacteremia CR-PsA	0.2	0.1	0.027	−13.5 (−24.0 to −1.6)	0.027
	(0.1 to 0.2)	(0.0 to 0.1)			
**Total Hospital Departments**					
Total Bacteremia	2.7	4.4	<0.001	2.4 (−1.7 to 6.6) up to December 2017	0.260
	(2.4 to 3.1)	(3.5 to 5.5)		43.2 (10.3 to 86.0) after December 2017	0.007
Total Bacteremia MDR Gram(+) and (−)	0.2 (0.1 to 0.4)	0.1 (0.1 to 0.2)	0.207	−10.3 (−24.2 to 6.2)	0.207
Total Bacteremia MDR Gram(−)	0.2 (0.1 to 0.3)	0.1 (0.1 to 0.2)	0.165	−11.1 (−24.8 to 5.0)	0.165
Total Bacteremia MDR Gram(+)	N/A	N/A	N/A	N/A	N/A
Total Bacteremia CR-Ac	N/A	N/A	N/A	N/A	N/A
Total Bacteremia CR-KlPn	0.1	0.0	0.445	−9.4 (−29.7 to 16.8)	0.445
	(0.0 to 0.2)	(0.0 to 0.1)			
Total Bacteremia CR-PsA	0.1	0.0	0.042	−22.9 (−39.9 to 0.9)	0.042
	(0.1 to 0.2)	(0.0 to 0.1)			
**Adults Clinic**					
Total Bacteremia	4.8	6.3	0.001	−0.6 (−5.4 to 4.5) up to November 2016	0.821
	(4.2 to 5.5)	(5.6 to 7.2)		15.5 (5.1 to 26.9) after November 2016	0.003
Total Bacteremia MDR Gram(+) and (−)	0.7 (0.5 to 0.9)	0.4 (0.3 to 0.6)	0.157	−7.0 (−15.8 to 2.8)	0.157
Total Bacteremia MDR Gram(−)	0.6 (0.4 to 0.8)	0.4 (0.3 to 0.5)	0.119	−7.4 (−15.8 to 2.0)	0.119
Total Bacteremia MDR Gram(+)	0.1 (0.0 to 0.2)	0.1 (0.0 to 0.1)	0.742	−4.3 (−26.3 to 24.3)	0.742
Total Bacteremia CR-Ac	0.1	0.1	0.980	−45.4 (−66.8 to −10.2) up to March 2016	0.017
	(0.1 to 0.3)	(0.1 to 0.4)		99.6 (9.4 to 264.2) after 0.024	0.024
Total Bacteremia CR-KlPn	0.1	0.1	0.614	56.3 (−6.7 to 161.7) up to February 2015	0.090
	(0.0 to 0.3)	(0.0 to 0.2)		−27.3 (−43.0 to −7.3) after February 2015	0.010
Total Bacteremia CR-PsA	0.3 (0.2 to 0.4)	0.1 (0.1 to 0.2)	0.031	−13.5 (−24.2 to −1.3)	0.031
**Adults Clinic Departments**					
Total Bacteremia	2.9 (2.6 to 3.4)	4.1 (3.6 to 4.7)	0.004	5.9 (1.9 to 10.1)	0.004
Total Bacteremia MDR Gram(+) and (−)	0.3 (0.2 to 0.6)	0.2 (0.1 to 0.4)	0.343	−8.0 (−22.6 to 9.3)	0.343
Total Bacteremia MDR Gram(−)	0.3 (0.2 to 0.5)	0.2 (0.1 to 0.3)	0.205	−10.0 (−23.6 to 5.9)	0.205
Total Bacteremia MDR Gram(+)	N/A	N/A	N/A	N/A	N/A
Total Bacteremia CR-Ac	N/A	N/A	N/A	N/A	N/A
Total Bacteremia CR-KlPn	0.1 (0.0 to 0.2)	0.1 (0.0 to 0.1)	0.495	−8.3 (−28.4 to 17.5)	0.495
Total Bacteremia CR-PsA	0.2 (0.1 to 0.4)	0.0 (0.0 to 0.1)	0.051	−21.8 (−38.9 to 0.1)	0.051
**Adults ICU**					
Total Bacteremia	18.2	32.8	<0.001	−3.8 (−15.2 to 9.1) up to February 2016	0.545
	(13.9 to 23.7)	(27.5 to 39.2)		28.6 (14.9 to 43.9) after February 2016	<0.001
Total Bacteremia MDR Gram(+) and (−)	1.9	2.3	0.678	34.5 (3.3 to 75.1) up to October 2015	0.028
	(1.2 to 3.0)	(1.2 to 4.2)		−18.9 (−38.1 to 6.3) after October 2015	0.130
Total Bacteremia MDR Gram(−)	2.5 (1.7 to 3.5)	3.3 (2.1 to 5.1)	0.392	4.9 (−6.0 to 17.1)	0.392
Total Bacteremia MDR Gram(+)	N/A	N/A	N/A	N/A	N/A
Total Bacteremia CR-Ac	0.8 (0.4 to 2.0)	1.8 (0.6 to 5.6)	0.256	−28.9 (−51.9 to 5.0) up to January 2017	0.086
				209.3 (46.7 to 552.3) after January 2018	0.003
Total Bacteremia CR-KlPn	0.3 (0.1 to 1.2)	0.5 (0.1 to 1.7)	0.635	137.8 (8.6 to 420.6) up to January 2015	0.030
				−28.4 (−51.8 to 6.3) after January 2015	0.098
Total Bacteremia CR-PsA	0.9 (0.4 to 1.8)	0.9 (0.4 to 2.1)	0.909	1.3 (−18.1 to 25.2)	0.909

ICU: intensive care unit; MDR: multidrug-resistant; CR: carbapenem-resistant; CR-Ac: carbapenem-resistant *Acinetobacter baumannii*; CR-KlPn: carbapenem-resistant *Klebsiella pneumoniae*; CR-PsA: carbapenem-resistant *Pseudomonas aeruginosa*; N/A: not applicable; EVSP: estimated value start period; EVEP: estimated value end period; CI: confidence interval; All estimates derived from Poisson regression models with robust standard errors, seasonality terms and linear or piecewise linear long-term trend: log(N) = β_0_ +β_1_t_−_ +β_2_t _+_ +β_3_ × sin(2πt/12) + β_4_ × cos(2πt/12) + β_5_ × sin(4πt/12) + β_6_ × cos(4πt/12) + log(patient-days) with N being the number of cases and t being time since study start in months (t_−_ and t_+_ piecewise linear time terms; when piecewise linear long term trend was not required a single time term was used). % Relative changes/year derived as [exp(12 × β_1_,_2_)−1] × 100%.

**Table 5:** Correlation of CAUTI and incidence of bacteremia in a hospital from January 2013 to December 2018.

CAUTI: Correlation with BACTEREMIA								
Incidence of Bacteremia/1000 Patient-Days	Per (n) Unit	Month 0	Month −1	Month −2	Month −3	IRR	95% CI	*p*-Value
Total Hospital Clinics								
Total Gram(+) and (−)	1		◊			0.81	0.75–0.87	<0.001
Total CR Gram(−)	0.1				◊	0.96	0.94–0.99	0.008
Total CR-Ac	0.1			◊		0.93	0.86–1.01	0.079
Total CR-KlPn	0.1				◊	0.87	0.82–0.92	<0.001
Total CR-PsA	0.1	◊				0.94	0.89–1.00	0.067
**Total hospital departments**								
Total Gram(+) and (−)	1		◊			0.78	0.71–0.86	<0.001
Total CR Gram(−)	0.1	◊				1.06	1.00–1.13	0.048
Total CR Gram(−)	0.1				◊	0.97	0.94–0.99	0.009
Total CR-KlPn	0.1				◊	0.90	0.85–0.95	<0.001
Total CR-PsA	0.1				◊	0.96	0.93–0.99	0.013
**Adults Clinic**								
Total Gram(+) and (−)	1	◊	◊			0.86	0.80–0.91	<0.001
Total CR Gram(−)	0.1				◊	0.98	0.96–1.00	0.042
Total CR-Ac	0.1			◊		0.95	0.91–1.00	0.029
Total CR-KlPn	0.1			◊	◊	0.85	0.80–0.90	<0.001
Total CR-PsA	0.1	◊				0.96	0.92–1.00	0.032
Total CR-PsA	0.1			◊		1.10	1.00–1.20	0.043
**Adults Clinic Departments**								
Total Gram(+) and (−)	1		◊			0.86	0.82–0.90	<0.001
Total CR Gram(−)	0.1	◊				1.04	1.00–1.08	0.060
Total CR Gram(−)	0.1				◊	0.98	0.96–1.00	0.027
Total CR-KlPn	0.1				◊	0.93	0.89–0.96	<0.001
Total CR-PsA	0.1				◊	0.97	0.95–1.00	0.038
**Adults ICU**								
Total Gram(+) and (−)	10		◊			1.09	0.99–1.20	0.086
Total CR Gram(−)	1	◊	◊	◊	◊	1.07	0.80–1.45	0.637
Total CR-Ac	1		◊			0.77	0.57–1.04	0.089
Total CR-Kl.Pn	1				◊	0.79	0.65–0.96	0.018
Total CR-PsA	1	◊				0.75	0.55–1.03	0.075
Total CR-PsA	1		◊			1.08	1.04–1.12	<0.001
Total CR-PsA	1			◊		1.24	1.11–1.38	<0.001

CAUTI: catheter-associated urinary tract infection; IRR: Incidence rate ratio; CI: Confidence Interval; ICU: intensive care unit; CR: carbapenem-resistant; CR-Ac: carbapenem-resistant *A.baumannii*; CR-KlPn: carbapenem-resistant *K. pneumoniae*; CR-PsA: carbapenem-resistant *P. aeruginosa*; ns: not-significant; Symbol ◊ denotes whether the association refers to the current month (month 0) value incidence of bacteremia, lagged values (months −1, −2, −3) or averaged values over more than one month. Incidence Rate Ratio (IRR) refers to increases incidence of bacteremia, denoted in column labeled “per (n) unit”. All estimates derived from Poisson regression models with robust standard errors, seasonality effects and spline terms of time: log(N) = β_0_ + β_1_V + β_2_S_1_(t) +β_3_S_2_(t) +β_4_S_3_(t) + β_5_ × sin(2πt/12) + β_6_ × cos(2πt/12) + β_7_ × sin(4πt/12) + β_8_ × cos(4πt/12) +log(catheter-days)with N being the number of cases, t being time since study start in months, S(t) being spline terms of t and V referring to the current month covariate (month 0) value, lagged values (months −1, −2, −3) or averaged values over more than one month. Incidence Rate Ratios derived as exp(n × β_1_) with n given in column labeled “per (n)”.

**Table 6:** Correlation of CAUTI and infection control interventions in a hospital from January 2013 to December 2018.

CAUTI: Correlation with Infection Control Interventions								
Infection Control Interventions	Per (n) Unit	Month 0	Month −1	Month −2	Month −3	OR	95% CI	*p*-Value
Total Hospital Clinics								
% isolations/admissions								ns
L of alcohol disinfectant sol/1000 patient-days								ns
L of scrub disinfectant sol/1000 patient-days	1			◊	◊	0.97	0.96–0.98	<0.001
L of all hand disinfectant sol/1000 patient-days	10				◊	0.85	0.76–0.95	0.004
**Adults Clinic**								
% isolations/admissions								ns
L of alcohol disinfectant sol/1000 patient-days								ns
L of scrub disinfectant sol/1000 patient-days	10			◊	◊	0.81	0.78–0.84	<0.001
L of all hand disinfectant sol/1000 patient-days	10	◊				1.05	0.99–1.11	0.075
L of all hand disinfectant sol/1000 patient-days	10				◊	0.89	0.82–0.97	0.006
**Adults Clinic Departments**								
% isolations/admissions								ns
L of alcohol disinfectant sol/1000 patient-days								ns
L of scrub disinfectant sol/1000 patient-days	10			◊	◊	0.89	0.86–0.93	<0.001
L of all hand disinfectant sol/1000 patient-days	10				◊	0.91	0.85–0.97	0.005
**Adults ICU**								
% isolations/admissions								ns
L of alcohol disinfectant sol/1000 patient-days	10	◊				1.10	1.02–1.19	0.012
L of scrub disinfectant sol/1000 patient-days	10		◊			0.79	0.65–0.96	0.018
L of all hand disinfectant sol/1000 patient-days	100	◊				1.72	1.11–2.66	0.016

CAUTI: catheter-associated urinary tract infection; IRR: incidence rate ratio; CI: Confidence Interval; ICU: intensive care unit; L: liter; sol: solution; ns: not-significant; Symbol ◊ denotes whether the association refers to the current month (month 0) value, lagged values (months −1, −2, −3) or averaged values over more than one month. Incidence Rate Ratios (IRR) refers to increases denoted in column labeled “per (n) units”. All estimates derived from Poisson regression models with robust standard errors, seasonality effects and spline terms of time: log(N) = β_0_ + β_1_V + β_2_S_1_(t) +β_3_S_2_(t) +β_4_S_3_(t) + β_5_ × sin(2πt/12) + β_6_ × cos(2πt/12) + β_7_ × sin(4πt/12) + β_8_ × cos(4πt/12) +log(catheter-days) with N being the number of cases, t being time since study start in months, S(t) being spline terms of t and V referring to the current month covariate (month 0) value, lagged values (months −1, −2, −3) or averaged values over more than one month. Incidence Rate Ratios derived as exp(n × β_1_) with n given in column labeled “per (n)”.

## Data Availability

The data that support the findings of this study are available on request from the corresponding author (H.C.M.). The data are not publicly available as data disclosure requires permission and ethical approval from Medical Ethical Committee of Athens Medical Center, Athens, Greece.
